# Activation of the rostral nucleus accumbens shell by optogenetics induces cataplexy-like behavior in orexin neuron-ablated mice

**DOI:** 10.1038/s41598-023-29488-x

**Published:** 2023-02-13

**Authors:** Shigetaka Kawashima, Fan Lou, Ikue Kusumoto-Yoshida, Liying Hao, Tomoyuki Kuwaki

**Affiliations:** 1 Department of Physiology, Graduate School of Medical and Dental Sciences, Kagoshima University, Kagoshima, Japan; 2 Department of Pharmaceutical Toxicology, School of Pharmacy, China Medical University, Shenyang, China; 3 Present Address: The First Affiliated Hospital of China Medical University, Shenyang, China

**Keywords:** Neuroscience, Circadian rhythms and sleep, Orexin

## Abstract

Cataplexy is one of the symptoms of type 1 narcolepsy, characterized by a sudden loss of muscle tone. It can be seen as a behavioral index of salience, predominantly positive emotion, since it is triggered by laughter in humans and palatable foods in mice. In our previous study using chemogenetic techniques in narcoleptic mice (orexin neuron-ablated mice), we found that the rostral nucleus accumbens (NAc) shell is needed for chocolate-induced cataplexy. In this study, we investigated whether a short-lasting stimulation/inhibition of the NAc by optogenetics led to a similar result. Photo-illumination to the NAc in the channel rhodopsin-expressing mice showed a higher incidence (34.9 ± 5.1%) of cataplexy-like behavior than the control mice (17.8 ± 3.1%, P = 0.0056). Meanwhile, inactivation with archaerhodopsin did not affect incidence. The episode duration of cataplexy-like behavior was not affected by activation or inactivation. Immunohistochemical analysis revealed that photo-illumination activated channel rhodopsin-expressing NAc shell neurons. Thus, activation of the NAc, whether transient (light stimulation) or longer-lasting (chemical stimulation in our previous study), facilitates cataplexy-like behaviors and contributes to the induction but not maintenance in them. On the other hand, our study's result from optogenetic inhibition of the NAc (no effect) was different from chemogenetic inhibition (reduction of cataplexy-like behavior) in our previous study. We propose that the initiation of cataplexy-like behavior is facilitated by activation of the NAc, while NAc-independent mechanisms determine the termination of the behavior.

## Introduction

Cataplexy is the main symptom of type 1 narcolepsy, a sleep disorder. The symptom is a sudden transient loss of muscle tone without impairment of consciousness^1^ triggered by strong emotions such as laughter, joke, or surprise in human^2^. The muscle atonia is observed in animal models with orexin receptor-2 gene mutant dog^3^, deficiency of orexin (also known as hypocretin) (*prepro-orexin* knockout mice)^4^, and orexin neuron-ablated (ORX-AB) mice^5,6^. These narcoleptic mice show cataplexy behavior caused by positive emotion with running wheel^7,8^, sweetened cereal^9^, and chocolate^8,10–12^. In addition, we previously showed that female encounters increased the number of cataplexy-like behaviors in male ORX-AB mice, and 85% of cataplexy-like behavior preceded ultrasonic vocalization (USVs) that is thought to reflect sexual excitation^11^. Thus, cataplexy is believed to be triggered by positive emotion in narcoleptic mice and can be used as a behavioral index of positive emotion.

The nucleus accumbens (NAc) is commonly known as the ventral striatum and a part of the mesolimbic structure called the reward pathway^13,14^. The NAc plays roles in positive emotional behavior such as motivation, drug abuse, alcohol, and rewards^15,16^. Blockade of dopamine receptors in the NAc reduces 50-kHz USVs as a marker of positive emotion induced by tactile stimulation of the skin^17,18^. The dorsal NAc shell activation of dynorphinergic neurons drives reward behavior and the ventral NAc shell drive aversion in mice^19^. Activation of projections from the basolateral amygdala to the NAc code positive valence in mice^20^. Furthermore, a previous study revealed the rostrocaudal segregation of emotional behavior in the NAc^21^. Activity in the rostral NAc increases when mice engage in behaviors associated with positive emotion, such as feeding. In contrast, activity in the caudal NAc increases with negative emotions such as defensive treading in rats^21^.

The NAc has also been considered a critical nucleus related to cataplexy with positive emotion. Narcolepsy patients show more activation of the NAc than healthy controls when reading humorous cartoon^22^. An fMRI study shows activation of the NAc during cataplexy in narcolepsy children^23^. Furthermore, our previous study shows that the rostral NAc shell markedly expresses the phosphorylated form of the extracellular signal-regulated kinase (pERK), one of the cellular activation markers, at the beginning of cataplexy^24^. In addition, activation/inhibition of the rostral NAc shell using designer receptors exclusively activated by designer drugs (DREADDs) increased/decreased the number of cataplexy during 12 h of observation period^24^. These findings indicate that the rostral NAc shell triggers or facilitates cataplexy.

In mice, DREADDs are available for long-term neuronal manipulation, from 5–10 min to approximately 9 h^25,26^, which has been used to study mood-related behavior^27–29^. Because cataplexy is a very short event (10-s to several min)^1,30^, long-term activation by DREADD cannot distinguish between the triggering role and facilitating role of elsewhere initiated signals. In our previous study, cataplexy-like behaviors repeatedly occurred when the DREADD system was thought to be active, indicating a facilitating role. In the same study, we also showed an increase in pERK immediately after cataplexy's beginning, indicating a triggering role. Therefore, there is a need to study the possible role of the NAc in cataplexy using high-time-resolution neuronal manipulation.

Emotion is a transient physiological response, and a change in an emotional state is caused by unconscious stimulation, often a short-term phenomenon such as msec-sec^31^. In contrast, the mood is characterized by a sustained emotional state regardless of external stimulation^32^. This temporal difference between emotion and mood may imply a difference between short-term and long-term neuronal mechanisms. This concept raised whether the NAc plays a similar role in emotion and mood. In our previous study, we tested the effect of relatively long-term neuronal manipulation of the NAc on cataplexy using the DREADDs system^24^. However, the possible effect of short-term neuronal manipulation of the NAc on cataplexy is not yet evaluated.

To address these questions, we investigated the effects of activation of the NAc on cataplexy-like behavior using channel rhodopsin 2 (ChR2). We also investigated the inactivation effects using archaerhodopsin (Arch) in ORX-AB mice. We used adeno-associated virus (AAV) to express ChR2-EYFP, Arch-EYFP, or EYFP under the control of calmodulin kinase IIα (CaMKIIα) promoter in the NAc. CaMKIIα promoter was selected because our previous study showed successful and restricted expression of the DREADD system in the NAc^24^, and CaMKIIα is restrictedly expressed in medium spiny neurons (MSN) of Dopamine D1 receptor expressing-type in the NAc^33^. We analyzed whether photo-illumination in the NAc affects the occurrence of cataplexy-like behavior. The latency was analyzed from the start of photo-illumination to the onset of cataplexy-like behavior. The duration of cataplexy-like behavior was also analyzed and compared between the on and off of photo-illumination.

## Results

### Optogenetic activation of the rostral NAc shell induces cataplexy-like behavior

**Figure 1:**
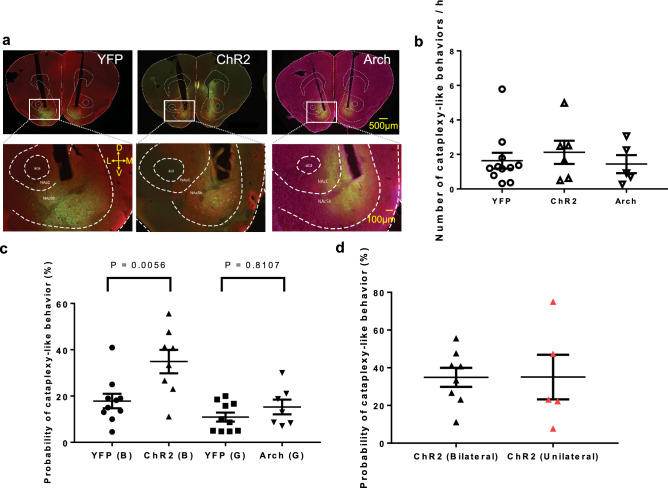
Expression of AAV in the rostral NAc shell and effect of photo-illumination in the rostral NAc shell on cataplexy-like behavior. (**a**) Typical examples of AAV spread (green) in the coronal section, including the rostral NAc shell from an AAV-YFP (left), an AAV-ChR2 (middle), and an AAV-Arch injected mouse (right). The section (right, AAV-Arch) was counterstained with NeuroTrace 530/615 red fluorescent Nissl stain to better detection of the region border. The lower images were enlargements of rectangles in the upper images. Scale bar = 500 µm (upper) and 100 µm (lower). Abbreviations: D, dorsal; V, ventral; M, medial; L, lateral; aca, anterior commissure; NAcC, nucleus accumbens core; NAcSh, nucleus accumbens shell. (**b**) The number of cataplexy-like behaviors per hour without photo-illumination. The possible effect of AAV expression in the rostral NAc shell on the frequency of spontaneous cataplexy-like behavior was examined without photo-illumination. No such effect was observed (One-way ANOVA, P = 0.7296, YFP n = 11, ChR2 n = 6, Arch n = 5). (**c**) Probability of cataplexy-like behavior induction by photo-illumination. The probability of cataplexy-like behavior in the ChR2 group (blue photo-illumination) was significantly larger than that in the YFP group (blue photo-illumination) (One-way ANOVA followed by Tukey’s multiple comparisons tests, P = 0.0056). The probability of cataplexy-like behavior in the Arch group (green photo-illumination) was not different from that in the YFP group (green photo-illumination). (YFP (B) n = 10, ChR2 (B) n = 8, YFP (G) n = 10, Arch (G) n = 7). (**d**) Probability of cataplexy-like behavior with bilateral or unilateral photo-illumination in ChR2-expressing mouse. The bilateral or unilateral photo-illumination showed no significant difference in the probability of cataplexy-like behavior (unpaired t-test, P = 0.9887, ChR2 (bilateral stimulation) n = 8, ChR2 (unilateral stimulation) n = 5). Data for bilateral stimulation were the replication of those shown in (**c**). Values from bilateral photo-illumination had a minor variation, although the average value was not different from that in unilateral photo-illumination. Therefore, we used bilateral photo-illumination in all of the other experiments. Horizontal bars represent the mean ± SEM in Fig. 1.

We previously demonstrated that hour-long activation and inhibition of the neurons in the rostral NAc shell increase and decrease the number of cataplexy episodes in narcolepsy model mice using the viral introduction of DREADDs^24^. However, the immediate effect of activation/inhibition of NAc on cataplexy was not yet tested. In this study, we used optogenetics to manipulate neuronal activity in a narrower time range than that using DREADDs. We tried to know the immediate effect since cataplexy-inducing emotional change may occur within a short time, ranging from seconds to minutes. In this experiment, we used three different adeno-associated viruses (AAV): AAV-YFP; yellow fluorescence, AAV-ChR2; channel rhodopsin 2, AAV-Arch; archaerhodopsin (Fig. 1a). The tip of the optical fiber track was observed above the rostral NAc shell (Fig. 1a). The AAV injected orexin neuron-ablated (ORX-AB) mice exhibited YFP/ChR2/Arch expression in the NAc (Fig. 1a).

Before photo-illumination experiments, we examined the possible effect of AAV expression in the rostral NAc shell on the frequency of cataplexy-like behavior without photo-illumination. When the number of spontaneous cataplexy-like behaviors per hour was compared among the three groups (AAV-YFP; 1.64 ± 0.46 times/h, n = 11, AAV-ChR2; 2.12 ± 0.67 times/h, n = 6, AAV-Arch; 1.44 ± 0.52 times/h, n = 5), there was no significant difference among them (Fig. 1b, F (2, 19) = 0.3206, One-way ANOVA, P = 0.7296, η^2^ = 0.03). Therefore, the expression of artificial receptors in the NAc by itself hardly affected the incidence of cataplexy-like behavior.

Chocolate bites did not always lead to cataplexy-like behavior; We observed 623 bites during the observation period of 124 h (total counts in 39 sessions), and only 73 (11.7%) bites were followed by cataplexy-like behavior within 3 min after bites. Therefore, chocolate had a facilitating but not a triggering effect to initiate cataplexy-like behavior.

We next examined whether photo-illumination affects the occurrence of cataplexy-like behavior (Fig. 1c). The probability of cataplexy-like behavior induction was defined as the percentage of the number of cataplexy-like behaviors occurrences within the photo-illumination period of 170 s in the number of photo-illumination. When the animal was awake and moving, 12–27 illuminations were applied to one animal during the observation period of up to 10 h. The result showed that the probability of photo-illumination-induced cataplexy-like behavior in the ChR2 group (34.9 ± 5.1%, n = 8) was significantly larger (Fig. 1c, Tukey’s test, P = 0.0056) than YFP group (blue photo-illumination, 17.8 ± 3.1%, n = 10) with large effect size (d = 1.42). On the other hand, the probability of cataplexy-like behavior was not different (Fig. 1c,  P = 0.8107, d = 0.7) between the Arch group (15.3 ± 3.2%, n = 7) and YFP group (green photo-illumination, 10.9 ± 2.0%, n = 10). These results suggested that activation of the rostral NAc shell using optogenetics induces cataplexy-like behavior in ORX-AB mice. However, we did not observe inhibition of cataplexy-like behavior with photo-illumination in Arch group mice.

We next examined whether bilateral photo-illumination is more effective than unilateral illumination (Fig. 1d). Unilateral photo-illumination of the NAc shell successfully induces cataplexy-like behavior. There was no significant difference (Fig. 1d, unpaired t-test, P = 0.9887, d = 0.01) in the probability of cataplexy-like behavior between bilateral photo-illumination (34.9 ± 5.1%, n = 8) and unilateral photo-illumination (35.1 ± 11.8%, n = 5). However, the coefficient of variation (CV) tended to be larger (P = 0.15 by F test) in the unilateral photo-illumination group (CV = 75.5%) than in the bilateral photo-illumination group (CV = 41.0%). Although there was no statistically significant difference between the average values in bilateral and unilateral stimuli, bilateral photo-illumination tended to have a minor variation. Therefore, we used bilateral photo-illumination in the following experiments.

### Optogenetic activation shortened the latency to start cataplexy-like behavior

**Figure 2:**
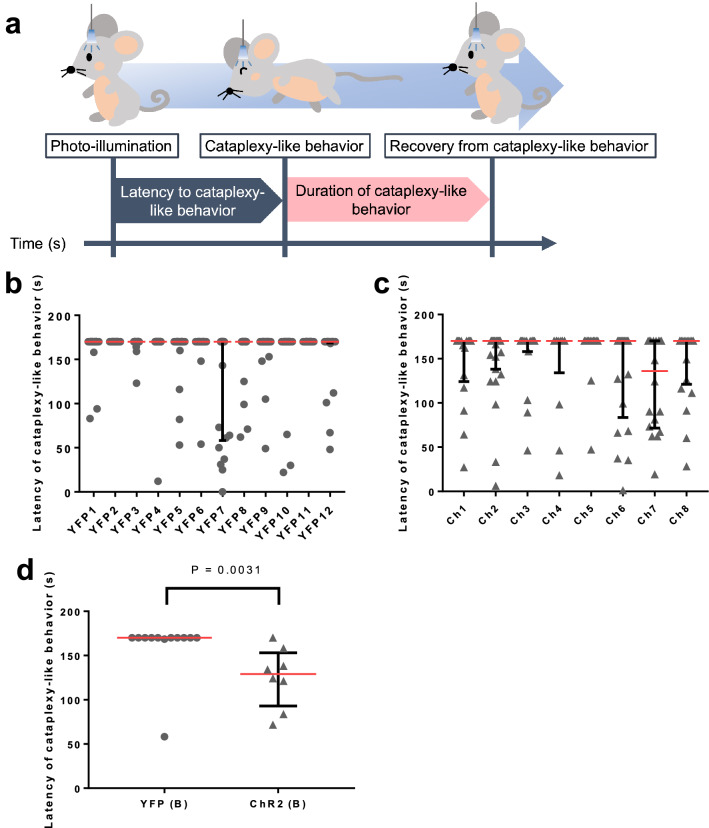
Latency of photo-illumination-induced cataplexy-like behavior. (**a**) Schematic explanation of the latency and duration of cataplexy-like behavior by photo-illumination on the rostral NAc shell. When cataplexy-like behavior was observed during photo-illumination, we call it “photo-illumination-induced cataplexy-like behavior." Otherwise, the cataplexy-like behavior was defined as “spontaneous cataplexy-like behavior." The latency of photo-illumination-induced cataplexy-like behavior was defined as the time from the start of photo-illumination to cataplexy-like behavior occurrence. When photo-illumination-induced cataplexy-like behavior was not observed, the latency was defined as 170 s with blue photo-illumination and 140 s with green photo-illumination. (**b**, **c**) The onset latency of cataplexy-like behavior in an individual mouse by blue photo-illumination for YFP and ChR2 expressing mouse. Each dot represents a single onset latency of cataplexy-like behavior in YFP group (**b**) and ChR2 group (**c**). 20–23 illuminations were applied to one animal in YFP group (**b**). 13–27 illuminations were applied to one animal in ChR2 group (**c**). Horizontal bars represent the median (red) with 25%-interquartile values (black). (**d**) Comparison of onset latency of cataplexy-like behavior between YFP and ChR2 expressing mouse. The dots show the 25-percentile value of the onset latency of the cataplexy-like behavior by photo-illumination in each animal. The latency was significantly shorter in the ChR2 group than that in the YFP group (Mann–Whitney U test, P = 0.0031, YFP (B) n = 12, ChR2 (B) n = 8). Two animals out of 12 animals in the YFP group never showed photo-illumination-induced cataplexy-like behavior.

We revealed that the probability of cataplexy-like behavior was significantly increased during optogenetic activation in the rostral NAc shell. However, we cannot distinguish between spontaneous occurrence and photo-illumination-induced occurrence of cataplexy-like behavior in our method. Therefore, we calculated the latency to start cataplexy-like behavior (Fig. 2a) to confirm further that the increase was induced by photo-illumination. Starting time of the spontaneous cataplexy should be randomly distributed within the photo-illumination period (170 s) if photo-illumination did not affect the occurrence of cataplexy-like behavior. To assess the latency of cataplexy-like behavior, we plotted all the latencies to start cataplexy behavior in each mouse (Fig. 2b and 2c). Each animal received 13–27 illuminations (Fig. 2b and c). When the photo-illumination did not induce the cataplexy-like behavior, the latency value was over 170 s. Since there is a cutoff value of 170 s, data points are not normally distributed. Therefore, we calculated the median and 25-percentile values in each mouse as the representative values (Fig. 2d). The 25-percentile value in the ChR2 group (129.0 [92.9, 153.0] s, n = 8, median and interquartile range in square bracket) was significantly shorter (Fig. 2d, Mann–Whitney U test, *P* = 0.0031, r = 0.59) than YFP group (blue photo-illumination, 170.0 [170.0, 170.0] s, n = 12). The result suggested that activation of the rostral NAc shell by photo-illumination on ChR2 facilitated the induction of cataplexy-like behavior.

### The duration of cataplexy-like behavior was not affected by artificial manipulation of the rostral NAc shell

**Figure 3:**
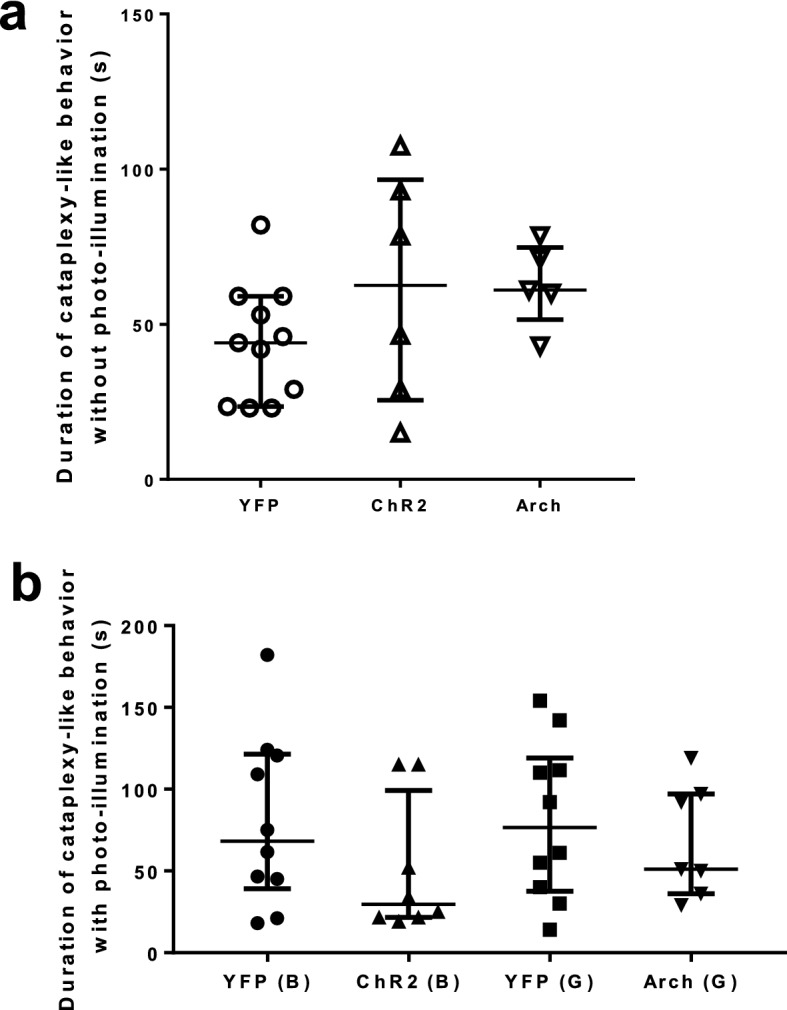
The rostral NAc shell did not regulate the duration of photo-illumination-induced cataplexy-like behavior. (**a**) Duration of cataplexy-like behavior without photo-illumination. The possible effect of AAV expression in the rostral NAc shell on the duration of spontaneous cataplexy-like behavior was examined without photo-illumination. No such effect was observed (Kruskal–Wallis test, P = 0.1923, YFP n = 11, ChR2 n = 6, Arch n = 5). Horizontal bars represent the median with the interquartile range. (**b**) Duration of cataplexy-like behavior with photo-illumination in the rostral NAc shell. The plot shows the median duration of cataplexy-like behavior with photo-illumination. Blue/green photo-illumination to ChR2/Arch group did not affect the duration of cataplexy-like behavior induced by photo-illumination (Kruskal–Wallis test, P = 0.4703, YFP (B) n = 10, ChR2 (B) n = 8, YFP (G) n = 10, Arch (G) n = 7). The data represent the median with an interquartile range.

We next examined whether there is a difference between the duration of artificially-induced cataplexy-like behavior and that of spontaneous ones. We calculated the duration of cataplexy-like behavior in three groups of mice (Fig. 3a). In a first step to exclude the possibility of an effect of AAV-expression per se on the duration of cataplexy-like behavior, we examined the duration of cataplexy-like behavior in AAV injected mice without photo-illumination. In this examination, the duration of cataplexy-like behavior was not significantly different among the YFP group (44.0 [23.5, 59.0] s, n = 11), ChR2 group (62.5 [25.5, 96.6] s, n = 6) and Arch group (61.0 [51.5, 74.8] s, n = 5) (Fig. 3a, Kruskal-Wallis test, *P* = 0.1923).

We next assessed the duration of photo-illumination-induced cataplexy-like behavior. Again, there was no difference in the duration of photo-illumination-induced cataplexy-like behavior among four groups (Fig. 3b, Kruskal–Wallis test, P = 0.4703) in the ChR2 group (blue photo-illumination, 29.5 [21.5, 99.3] s, n = 8), YFP group (blue photo-illumination, 68.3 [39.0, 121.4] s, n = 10), Arch group (green photo-illumination, 51.0 [36.0, 97.0] s, n = 7), and YFP group (green photo-illumination, 76.5 [37.5, 119.1] s, n = 10). In addition, the overall average values (~ 50 s) were similar between those with (Fig. 3b) and without (Fig. 3a) photo-illumination conditions. The result suggested that the rostral NAc shell activation and inactivation did not regulate the duration of cataplexy-like behavior.

### The rostral NAc shell did not regulate the duration of spontaneous cataplexy-like behavior

**Figure 4:**
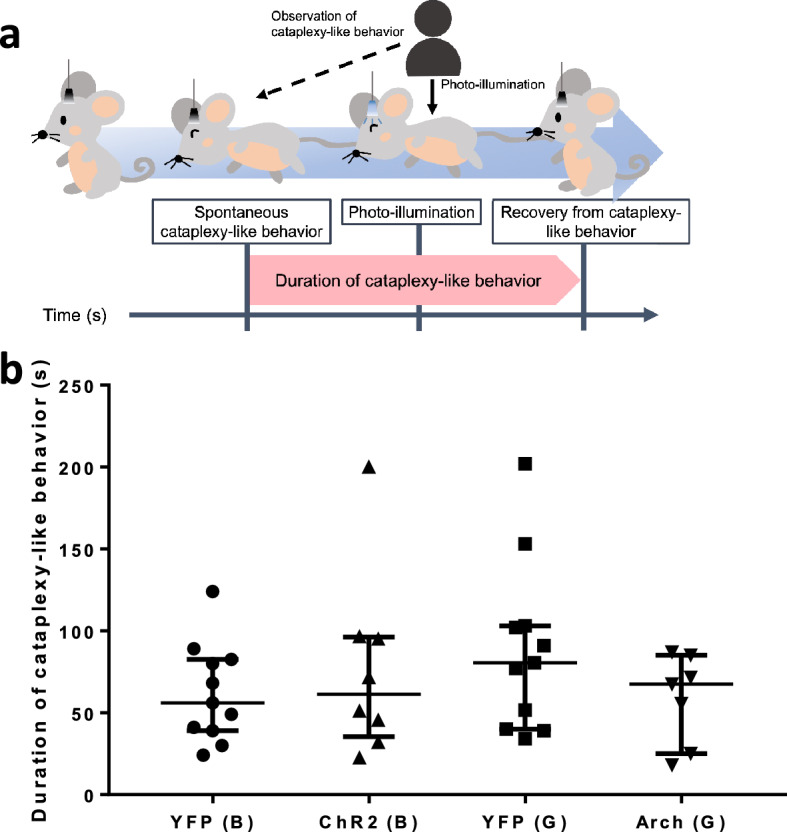
Effect of activation/inactivation in the rostral NAc shell on the spontaneous cataplexy-like behavior. (**a**) Schematic explanation of observation of photo-illumination on the spontaneous cataplexy-like behavior. In some experimental sessions, the mouse was given immediate (15.7 ± 1.3 s) photo-illumination when the beginning of spontaneous cataplexy-like behavior was observed. The possible modifying effect of blue/green photo-illumination on the spontaneous cataplexy-like behavior was examined. (**b**) Duration of cataplexy-like behavior when the mouse was given photo-illumination at the beginning of spontaneous cataplexy-like behavior. The plot showed the median duration of cataplexy-like behavior when the mouse was given photo-illumination at the beginning of spontaneous cataplexy-like behavior. Blue/green photo-illumination in ChR2/Arch group did not affect the duration of spontaneous cataplexy-like behavior (Kruskal–Wallis test, P = 0.4985, YFP (B) n = 11, ChR2 (B) n = 8, YFP (G) n = 11, Arch (G) n = 7). The data represent the median with an interquartile range.

Next, we examined whether artificial activation and inactivation would affect the duration of ongoing spontaneous cataplexy-like behavior. For this purpose, photo-illumination was given just after the confirmation of spontaneous cataplexy-like behavior had started (Fig. 4a). The observer waited for 10 s to confirm the cataplexy-like behavior and then started photo-illumination. The ChR2 group (61.3 [35.4, 96.1] s, n = 8) showed no difference from the blue photo-illuminated YFP group (56.0 [39.0, 82.5] s, n = 11). Also, there was no difference between the Arch group (67.5 [25.0, 85.0] s, n = 7) and green photo-illuminated YFP group (80.5 [40.0, 103.0] s, n = 11) (Fig. 4b, Kruskal–Wallis test, P = 0.4985). Thus, we did not observe any difference in the duration of cataplexy-like behavior by optogenetic manipulation of the rostral NAc shell (Figs. 3b and 4b).

### Histological examination revealed activation of ChR2-expressing neurons by photo-illumination

**Figure 5:**
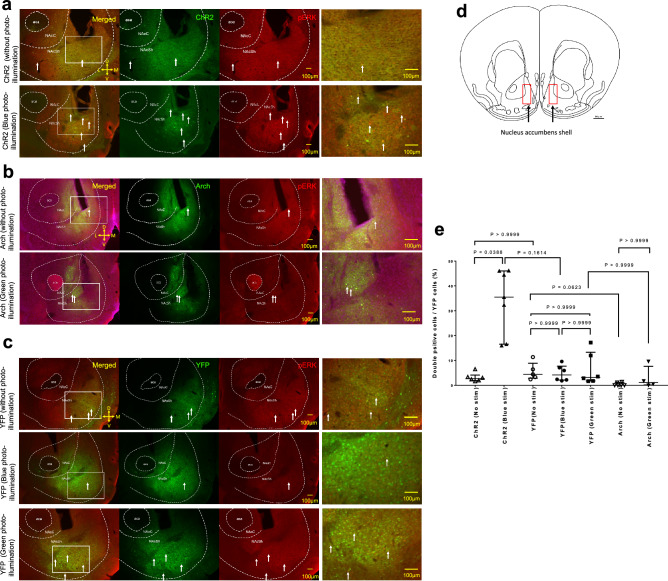
pERK immunostaining in the rostral NAc shell and percentage of double-positive cells in the rostral NAc shell. (**a**–**c**) Typical photographs from a brain of (from top to bottom); ChR2 mouse without photo illumination, ChR2 mouse with blue photo illumination, Arch mouse without photo illumination, Arch mouse with green photo illumination, YFP mouse without photo illumination, YFP mouse with blue photo illumination, and YFP mouse with green photo illumination. The left column is merged images and the middle left column is a green channel representing ChR2 (in a), Arch (in b), and YFP (in c). The middle right column is a red channel representing pERK and the rightmost channel is enlarged images shown in a white rectangle in the leftmost column. White arrows indicate double-positive cells in the NAc shell. Scale bar = 100 µm. Abbreviations: D, dorsal; V, ventral; M, medial; L, lateral; aca, anterior commissure; NAcC, nucleus accumbens core; NAcSh, nucleus accumbens shell. (**d**) Schematic diagram of the rostral NAc shell. The schematic diagram shows the location of the rostral NAc shell taken from the mouse brain atlas. The red rectangles were counted in the area (400 × 1000 μm) of AAV-YFP, AAV-ChR2, or AAV-Arch cells and pERK cells, including the rostral NAc shell. Scale bar = 500 µm. (**e**) The percentage of double-positive cells (pERK-positive and YFP-positive cells / YFP-positive cells) in the rostral NAc shell. Kruskal–Wallis test showed a significant difference among the groups (P = 0.0001, ChR2 (No stim) n = 6, ChR2 (Blue stim) n = 7, YFP (No stim) n = 5, YFP (Blue stim) n = 6, YFP (Green stim) n = 6, Arch (No stim) n = 6, Arch (Green stim) n = 4). A post hoc test revealed a significant difference between ChR2 (No stim) and ChR2 (Blue stim). *P* values from Dunn's test were indicated in the figure. Horizontal bars represent the median with the interquartile range.

To examine the possible effect of photo-illumination on cellular activation, we examined the phosphorylated form of the extracellular signal-regulated kinase (pERK) in the NAc (Fig. 5a–c).

To compare the degree of activation by photo-illumination, we calculated the percentage of double-positive (pERK-YFP) cells in YFP cells distributed in the rostral NAc shell (Fig. 5d) without photo-illumination or just after the end of photo-illumination. Kruskal–Wallis test exhibited significant differences among the groups (P = 0.0001). Dunn's test revealed that there was a significant difference between the no-illuminated ChR2 group (2.7 [1.9, 4.1] %, n = 6) and blue light-illuminated ChR2 group (35.5 [16.6, 46.1] %, n = 7) (Fig. 5e, P = 0.0388, r = 0.83). However, we found no difference (Fig. 5e, Dunn’s test, P > 0.9999, r = 0.41) in the percentage of double-positive (pERK-YFP) cells between no illuminated Arch group (0.0 [0.0, 1.2] %, n = 6) and green light illuminated Arch group (1.1 [0.1, 7.6] %, n = 4) nor among three (no illumination, blue and green light illumination) YFP groups. The result showed that blue light illumination to the ChR2 group activated the neurons in the rostral NAc shell and suggested that the activation of these cells had induced cataplexy-like behavior in the ORX-AB mice.

## Discussion

We have previously shown using the DREADD system that activation of the NAc shell increases the incidence of cataplexy-like behavior during the 12 h of observation period and inactivation decreases it. To further characterize the role of the rostral NAc shell in cataplexy-like behavior, we manipulated the activity of the rostral NAc shell using a short-term stimulation/inhibition by optogenetics. We hypothesized that there would be an overall similarity between the results from DREADD and those from optogenetic manipulations. However, there would be some differences since feelings can be divided into emotion and mood depending on the time scale. They may have both common and different brain mechanisms. In particular, we analyzed the probability of occurrence, the latency, and the duration of cataplexy-like behavior. We showed that activation of the rostral NAc shell by ChR2 increased the probability of cataplexy-like behavior. The latency of cataplexy-like behavior in the ChR2 group was significantly shorter than in the YFP group. We compared the 25-percentile value but not the median value (Fig. 2d) because, in 7 out of 8 ChR2-animals, the probability of cataplexy-like behavior did not exceed 50% (Fig. 1c). Hence, median latency equaled the cutoff value of 170 s (Fig. 2c). The duration of cataplexy-like behavior was not different between photo-illumination-induced cataplexy-like behavior and spontaneous cataplexy-like behavior. On the other hand, short-term inactivation of the rostral NAc shell did not affect the occurrence, latency, or duration of cataplexy-like behavior. These results suggest that transient activation of the rostral NAc shell triggered an initiation of cataplexy-like behavior and did not affect the duration of it when the behavior once started. Contrary to our expectation, transient inactivation of the rostral NAc shell did not show an inhibitory role in cataplexy-like behavior.

### Technical consideration

AAV injection and implantation of optical fiber in the rostral NAc shell did not distort cataplexy-like behavior for the following reasons. First, the tracking tip of the optical fiber was correctly positioned above the rostral NAc, where AAV expression was observed (Fig. 1a). AAV did not affect the frequency of spontaneous cataplexy-like behavior (Fig. 1b) or the duration of cataplexy-like behavior without photo-illumination (Fig. 3a).

### Transient activation of the rostral NAc shell induces cataplexy-like behavior

An imaging study indicated that emotional humor stimulation increases the activity of the NAc in narcolepsy patients compared with healthy controls^22^. Our previous study showed that activation of the rostral NAc shell with DREADDs increased the number of cataplexy events in the 12 h dark (active) period^24^. In addition, the expression of pERK in the rostral NAc shell was higher (205%) than in control at the beginning of cataplexy^24^. In agreement with previous studies, we showed here that the probability of photo-illumination-induced cataplexy-like behavior in the ChR2 group (blue photo-illumination) was significantly larger than that in the YFP group (blue photo-illumination) (Fig. 1c).

From the behavioral point of view, it is impossible to distinguish between spontaneous and photo illumination-induced cataplexy-like behaviors. Thus, the probability of cataplexy-like behavior in Fig. 1c represents the sum of spontaneous and photo illumination-induced cataplexy-like behaviors during the photo illumination period of 170-s. Since spontaneous cataplexy-like behaviors were observed ~ 2 times/h (Fig. 1b), an expected number of spontaneous cataplexy-like behaviors during the period of 170-s could be calculated as ~ 0.1, corresponding to 10% of probability. The value is similar to those in YFP and Arch groups (Fig. 1c) and far lower than the ChR2 group. Therefore, increased probability in the ChR2 group could be estimated as photo illumination induced. Shorter onset latency in the ChR2 group than in the YFP group (Fig. 2d) also supports this interpretation.

Further, we found a significantly higher percentage of double-positive cells (pERK-YFP) in the ChR2 group with photo-illumination than that without photo-illumination (Fig. 5e). These results suggest that transient activation of the rostral NAc is enough to induce cataplexy-like behavior.

### Transient inactivation of the rostral NAc shell does not prevent cataplexy-like behavior

Our previous study showed that inhibition of the rostral NAc shell with DREADDs decreased the occurrence of chocolate-induced cataplexy^24^. We expected a similar result in this study. However, we did not find a significant difference in the probability of cataplexy-like behavior between the Arch group (green photo-illumination) and the YFP group (green photo-illumination) (Fig. 1c). The length of inhibition might cause this discrepancy. Inhibition of GABAergic neurons of the central nucleus of the amygdala with DREADDs reduced cataplexy in the presence of chocolate and running wheel in orexin knockout mice^12^. These previous studies showed that chemogenetic continuous neuronal inhibition decreased cataplexy.

A previous report showed that activation of serotonin neurons in the dorsal raphe nucleus reduced the duration and the number of cataplexy-like behavior by stabilized step-function opsin (SSFO), a variant of ChR2, in orexin-ataxin3 mice^34^. In turn, optogenetic stimulation of serotonin fibers in the amygdala reduced the total time and the number of cataplexy-like episodes during 4-h of chocolate feeding^34^. Furthermore, SSFO maintained the stability of the activated state of targeted cells for more than 30 min^35^. In addition, serotonin and serotonin agonists decreased glutamate-evoked firing in the NAc^36^. All these studies utilized manipulations inducing continuous inhibition. In this study, however, we applied transient and intermittent photo-illumination to the Arch group in the rostral NAc shell for neuronal inhibition. After photo-illumination, the neuronal silencing began within hundreds of ms^37^ and recovered to the baseline firing rate within approximately 4 s^37^. Meanwhile, DREADDs caused inhibition of neurons in the time frame of seconds, minutes, and hours^38^. This different length of inhibition suggests a mechanism for the prevention of cataplexy. Thus, continuous but not transient inhibition in the rostral NAc shell might prevent the occurrence of cataplexy.

### Activation/inactivation of the rostral NAc did not regulate the duration of cataplexy-like behavior

We calculated the duration of cataplexy-like behavior to understand the functional role of the rostral NAc shell in determining it. As a result, the duration of the cataplexy-like episode did not change by activation or inhibition of the NAc (Fig. 3b).

In addition, the duration of cataplexy-like behavior was not modified by photo-illumination given at the beginning of the spontaneous one (Fig. 4b). These data collectively show that transient change in the activity of the rostral NAc shell does not affect the maintenance of cataplexy-like behavior.

On the other hand, we showed that transient neuronal activation of the rostral NAc shell did induce cataplexy-like behavior. The results indicate that the rostral NAc shell plays a role in causing but not maintaining cataplexy-like behavior. This difference suggests that the mechanism of the transient activation of the rostral NAc shell to induce cataplexy is different from the regulatory mechanism of maintaining it.

## Limitations

In this study, the percentage of double-positive cells (pERK-YFP) in YFP cells in the rostral NAc shell of the Arch group with photo-illumination was not different compared to that without photo-illumination (Fig. 5e).

Nevertheless, the NAc should be inactivated in this study for several reasons. First, we used the same photo-illumination parameter in our previous study^39^. Photo-inactivation of the medullary raphe serotonergic neurons reduced stress-induced tachycardia and tachypnea. However, it did not affect basal values of the heart rate and respiratory frequency^39^. Photo-inactivation also inhibited the stress-induced increase of pERK-positive cells, whereas basal expression did not reduced^39^. Therefore, a possible explanation of the current result may be that inhibition with Arch in the rostral NAc shell may not decrease basal expression of neuronal activity markers already expressed in the rostral NAc shell, namely, the “floor” effect. Although we added chocolate in the recording chamber, it had a facilitating but not a triggering effect to initiate cataplexy-like behavior. Since we could not predict the timing of cataplexy-like behavior, it was impossible to adjust the timing of optogenetic inhibition just before the cataplexy-like behavior would occur. Second, in another previous study, we showed that activation of the NAc using DREADDs increased the number of c-Fos, another neuronal activity marker, -positive cells in the rostral NAc shell. In contrast, inhibition did not change the number of c-Fos-positive cells^24^. Meanwhile, inhibition by DREADDs decreased the number of cataplexy^24^. Thus, histological data was only sometimes sensitive as physiological data. Nevertheless, further study is needed to understand better the expression of pERK in the rostral NAc shell using the inhibition of neuronal manipulation.

We have not examined the cell type specificity of the NAc shell in this study. Although the CaMKIIα promoter was used in this study and the restricted expression of CaMKIIα in the D1-type MSN in the NAc is reported^33^, other cell type-specific promoters should be examined in future studies. Furthermore, direct excitatory input from orexin neurons to D2-type MSNs in the NAc is indispensable for innate risk-avoidance behavior^40^, probably associated with negative but not positive emotion. Thus, the valence-related issue also should be studied future.

## Conclusion

In summary, we showed that short-term neuronal manipulation of the rostral NAc shell induced cataplexy-like behavior and facilitated the expression of the cataplexy-like behavior. We did not observe the prevention of cataplexy-like behavior by transient inactivation of the rostral NAc shell. These results were partially consistent with our previous study using long-term neuronal manipulation in the rostral NAc shell. We propose that once cataplexy-like behavior is triggered by activation of the NAc, termination of the behavior is determined by NAc-independent mechanisms.

## Materials and Methods

### Ethics approval

All experiments were performed at Kagoshima University following ARRIVE guidelines and the guiding principles for the care and use of animals in the field of physiological sciences published by the Physiological Society of Japan (2015). Protocols were reviewed and approved by the Experimental Animals Research Committee of Kagoshima University (MD17105).

### Animals

The male orexin neuron-ablated (ORX-AB) mice (22–36 g, n = 73) were used as the animal model of narcolepsy and were at least 12 weeks old at the virus injection. A method for selective ablation of orexin neurons has previously been reported^41^. In short, orexin-tTA mice, which express tetracycline transactivator (tTA) exclusively in orexin neurons under the control of the human prepro-orexin promoter^41^, were bred with tetO diphtheria toxin A fragment (DTA) mice (B6.Cg-Tg (tetO DTA) 1Gfi/J, The Jackson Laboratory) to generate orexin-tTA; tetO DTA mice. In these double transgenic mice (called ORX-AB in this paper), doxycycline is removed from their chow starting from birth, so by 4 months of age, almost all (> 97%) of the orexin neurons were ablated^41^. The original pair was a generous gift from Prof. Yamanaka at Nagoya University and were bred in Kagoshima University’s facility. Ablation of orexin neurons was confirmed, as was the case in our previous study^11,24,42^. Animals were maintained at room temperature (23 ± 1 °C) and housed on a 12-h light/dark cycle (lights on at 7:00 and off at 19:00). Mice had food and water available ad libitum. Mice were housed individually after adeno-associated virus (AAV) injections. To observe cataplexy-like behavior in the daytime for experimenters, we put the mice on a reversed light/dark cycle after the surgery (19:00 light on and 7:00 off). Behavior experiments were performed during the dark period because mostly cataplexy occurs during the dark period in narcoleptic mice^10,11^.

### Stereotaxic injection of AAV into the NAc

AAV injection surgeries were performed under inhalation anesthesia with 3% isoflurane using a stereotaxic instrument (ST-7, Narishige, Tokyo, Japan). The mice were given an analgesic (buprenorphine, 0.05 mg/kg) and an antibiotic (penicillin G, 40,000 U/kg) s.c. after the mice were anesthetized. Mice used Vaseline on their eyes to protect against drying. After removing the hair, the scalp was sterilized with 70% alcohol. Next, a glass micropipette (2–000-001, Drummond Scientific, Broomall, PA, USA) made with a puller (PC-10, Narishige, Tokyo, Japan) and a tip diameter of 50 µm was filled with one of the following AAVs, a gift from Karl Deisseroth. AAV (serotype 5)-CaMKIIα-hChR2(H134R)-EYFP (University of North Carolina vector core, Lot# AV4316(I + J)rp, 4.7 × 10^12^ virus molecules/ml), AAV5-CaMKIIα-eArchT3.0-EYFP (Lot# AV4883D, 3.8 × 10^12^ virus molecules/ml), or AAV5-CaMKIIα-EYFP (Lot# AV4808I, 3.6 × 10^12^ virus molecules/ml). The mice were injected with one type of AAV in bilateral NAc. The micropipette tips were placed on NAc (anterior 1.8 mm, lateral ± 0.8 mm, ventral 4.85 mm from bregma). All coordination was referenced from Paxinos and Franklin’s mouse brain atlas^43^. Next, the virus (300 nl) was injected (over 10 min on each side) with a gas-pressure microinjector (BJ-110, BEX, Japan) that attach to a micropipette with a silicone tube. After the withdrawal of the pipette, antibiotic ointment (bacitracin, 250 U/g: fradiomycin sulfate, 2 mg/g) was applied to the skull. Next, the mice were placed on a 37 °C warm plate after surgery for recovery.

### Implantation of optical fibers

Five weeks after the injection of AAV into the NAc, mice were implanted with glass optical fiber (0.2 mm in core diameter, 0.22 NA; KYOCERA, Kyoto, Japan). Mice were anesthetized with 3% isoflurane inhalation and were fixed on a stereotaxic device. The sterilization and analgesic procedures were identical to the AAV injection described above. Holes were drilled for anchoring screws on the left and right parietal bone, one on each side. The skull was cleaned with H_2_O_2_ and was applied to dental cement (Super-Bond, SUN MEDICAL, Shiga, Japan) except for the drilled hole. The optical fibers attached micromanipulator (SMM-100, Narishige, Tokyo, Japan) were implanted into NAc (anterior 1.8 mm, lateral ± 1.65 mm, ventral 4.12 mm, angled medial 10° from bregma). The optical fibers were fixed with dental acrylic (QUICK RESIN A, SHOFU INC., Kyoto, Japan). After surgery, mice were placed in reversed light/dark cycle box (7:00 light off, 19:00 light on) at least one week before behavior experiments.

### Observation of cataplexy-like behavior

Before the experiment day, mice were transferred from the home cage to an experimental chamber (45 cm × 33 cm × 24 cm) in a soundproof box with foods (regular chow), water, and chocolates (HERSHEY’S KISSES milk chocolate, The Hershey Company, USA.) to facilitates incidence of cataplexy^10^. We performed video recording during the dark period (active phase of mice) with a video camera (CBK21AF04, The Imaging Source Asia, Taipei, Taiwan) and observed mice behavior from outside of the soundproof box by a computer monitor using a video acquisition system in LabChart (ADInstruments, New Zealand). During the dark, the chamber was illuminated with a far infrared lamp (940 nm, SA2-IR, World Musen, Hong Kong). On the photo-illumination experimental day, mice were attached optical cable (Logos, Ibaraki, Japan) on ferrule (KYOCERA) to a laser device. The experiment started after the mice were replaced in the experimental chamber for over 20 min for acclimatization.

Cataplexy-like behavior was defined as established criteria for mice that were several observable features: (1) The mice were lasting immobility for at least 10 s with muscle weakness, (2) atonia was determined to be occurring when mice were in a prone position with their head and belly down in the bedding with their limbs and tail typically situated straight out from the trunk, (3) There must be at least 40 s of active wakefulness (moving) preceding atonia episode, (4) Duration of cataplexy-like behavior was limited to a maximum of 240 s to distinguish cataplexy-like behavior from sleep. In addition, we paid special attention not to include behavioral arrest other than cataplexy-like behavior such as sleep, freezing, and delta sleep attack (a peculiar sleep attack in the ORX-AB mice^44^) by observing collapsing body posture; the distance from nose to tail is longer than that in the standing and sleeping postures.

### Observation of photo-illumination-induced cataplexy-like behavior

Cataplexy-like behavior was measured during the dark period (from 8:00 to 19:00). Mice were given photo-illumination when awake and moving (walking, running, eating, drinking, digging, or grooming) or during cataplexy-like behavior as judged by real-time video monitoring on the screen outside of the soundproof box. During the experimental period of up to 10 h, 12–27 illuminations were applied to one animal. Each illumination was separated by at least 5 min. The photo-illumination-induced cataplexy-like behavior was defined as those that occurred during photo-illumination (170 s for blue light and 140 s for green light). Duration of photo-illumination-induced cataplexy-like behavior was considered the time between the onset of cataplexy-like behavior and recovery from atonia (Fig. 2a). The latency of cataplexy-like behavior was defined as the time from the start of photo-illumination to the beginning of cataplexy-like behavior (Fig. 2a).

### Observation of spontaneous cataplexy-like behavior without photo-illumination

The cataplexy-like behavior without photo-illumination was observed except for a period of photo-illumination.

### Photo-illumination

The optical fiber was connected to a 473 nm or 532 nm diode pumped solid state (DPSS) laser (473 nm laser: BL473T8-100FC, Shanghai Laser & Optics Century Co., Ltd., China, 532 nm laser: GL532T3-300FC, Shanghai Laser & Optics Century Co., Ltd., China) with optical cable located on outside the soundproof box via rotary joint (FRJ_1 × 1_FC-FC, Doric Lenses, Quebec, Canada). The laser was controlled with a stimulator (SEN-3301, NIHON KOHDEN, Tokyo, Japan) and adjustable power supply (473 nm laser: ADR-800A, Shanghai Laser & Optics Century Co., Ltd., China, 532 nm laser: ADR-700A, Shanghai Laser & Optics Century Co., Ltd., China). Before each behavior experiment, the final output of laser power was adjusted to 8 mW by using an optical power meter (PM20, Thorlabs, Newton, New Jersey, USA). To activate ChR2, a 473 nm (10 Hz) laser was provided for 20 s, which has a 10 s interval between each photo-illumination, and six times photo-illumination was considered one set photo-illumination. To activate Arch, a 532 nm (continuous) laser was provided for 15 s, which has a 10 s interval between each photo-illumination. Six times photo-illumination was considered as one set of photo-illumination. AAV-YFP mice received illumination with two conditions: Bule light (473 nm) as the control for the ChR2 group and green light (532 nm) as the control for the Arch group on a different experimental day. The method of photo-illumination was modified based on Konadhode et al*.*, 2013 and our previous study^39,45^.

## Immunohistochemistry

We examined whether AAV-infected neurons were activated by photo-illumination by immunostaining the phosphorylated form of the extracellular signal-regulated kinase (pERK) in the rostral NAc shell.

After photo-illumination, the mice were anesthetized with urethane (1.8 g/kg, i.p.) and transcardially perfused. Perfusion started within 7 min of the termination of photo-illumination. First, the mice were perfused with 20 ml of phosphate-buffered saline (PBS, 0.01 M, pH 7.4), followed by 20 ml of fixative, 4% paraformaldehyde (PFA) in 0.01 M PBS solution. The head was post-fixed at 4 °C overnight, immersed in 30% sucrose in PBS for 2 days, and the brains were removed from the skull. The brains were sliced into 40 µm sections with a vibratome (SuperMicroSlicer Zero1, DOSAKA EM, Kyoto, Japan). The slices were used for immunostaining every fourth section. The immunostaining procedure was performed at room temperature and in a dark box. First, the brain slices were washed with PBS and immersed in a blocking solution (1% normal horse serum and 0.3% Triton-X in 0.01 M PBS) for 30 min. Next, the brain slices were immersed in primary antibody diluted with blocking solution, anti-green fluorescence protein (GFP) rat antibody (04,404–84, Nacalai Tesque Inc., 1/1000), or anti-pERK rabbit antibody (4370S, Cell Signaling Technology, 1/1000) for overnight. Then the slices were immersed in secondary antibody; anti-rat IgG Cy2 (712–546-153, Jackson ImmunoReserch Inc., 1/1000) and anti-rabbit IgG-biotin complex (711–065-152, Jackson ImmunoReserch Inc., 1/300) for 2-h. Finally, the slices were immersed in streptavidin-conjugated Alexa-568 diluted with PBS (S11226, Invitrogen, 1/500) for 90 min. Sections of mice injected with AAV-Arch were stained with NeuroTrace 530/615 Nissl Stain (N21482, Thermo Fisher Scientific) at 1/100 for 20 min to identify brain structures.

## Cell number quantitative counting in the rostral NAc shell

The quantitative analysis method was reported by Su et al.^24^. In brief, we placed a counting box (400 × 1000 μm) bilaterally over the rostral (1.42 mm^24^ rostral to bregma^41^) NAc shell, just above the ventral pallidum and just lateral to the island of Calleja^43^(Fig. 5d).

### Statistics

Statistical analyses were performed using Prism software v.7 (GraphPad Software, San Diego, CA, USA). For the data in the duration of the cataplexy-like behavior experiment and histological analysis in the rostral NAc shell, we used a Kruskal-Wallis test followed by Dunn’s test. For the data on the probability of cataplexy-like behavior with bilateral stimulation and unilateral stimulation, we used Student’s t-test. We used a Mann-Whitney U test for the data on the latency of cataplexy-like behavior. For analysis of the number of cataplexy-like behaviors and the probability of cataplexy-like behavior, we used a one-way ANOVA followed by Tukey’s multiple comparison test. P <  0.05 was considered statistically significant. We also calculated the effect size for comparing two groups as Cohen’s d or r and three or more groups as η^2^. The effect size was considered as large when d > 0.8, r > 0.5, η^2^ > 0.14, medium when d > 0.5, r > 0.3, η^2^ > 0.06, and small when d > 0.2, r > 0.1, η^2^ > 0.01. Data are expressed as the mean ± SEM or median and quartile range.

## Supplementary Information

Supplementary Information.

## Data Availability

Summary statistics are available in the article. In addition, the raw data supporting this study's findings are available in the supporting file.
